# Haplotype association analysis of North American Rheumatoid Arthritis Consortium data using a generalized linear model with regularization

**DOI:** 10.1186/1753-6561-3-s7-s32

**Published:** 2009-12-15

**Authors:** Wei Guo, Chin-yuan Liang, Shili Lin

**Affiliations:** 1 Department of Statistics, The Ohio State University, Columbus, Ohio 43210 USA

## Abstract

The Genetic Analysis Workshop 16 rheumatoid arthritis data include a set of 868 cases and 1194 controls genotyped at 545,080 single-nucleotide polymorphisms (SNPs) from the Illumina 550 k chip. We focus on investigating chromosomes 6 and 18, which have 35,574 and 16,450 SNPs, respectively. Association studies, including single SNP and haplotype-based analyses, were applied to the data on those two chromosomes. Specifically, we conducted a generalized linear model with regularization (rGLM) approach for detecting disease-haplotype association using unphased SNP data. A total of 444 and 43 four-SNP tests were found to be significant at the Bonferroni corrected 5% significance level on chromosome 6 and 18, respectively.

## Background

Genetic Analysis Workshop (GAW) 16 Problem 1 involves studies designed to investigate genetic risk factors for rheumatoid arthritis (RA). The data are the initial batch of whole-genome scans for the North American Rheumatoid Arthritis Consortium (NARAC) cases (n_1 _= 868) and controls (n_2 _= 1194). The HLA region on 6p21 has been implicated by numerous studies and there is consistent evidence that the DR alleles contribute to disease risk [1]. The region on chromosome 18q has also shown evidence for linkage to RA in U.S. and French linkage scans [2,3]. Therefore, we focused our association study on these two chromosomes.

Recent advances in molecular technology lead to the availability of a large number of SNPs, and there are increasing interest in association studies involving haplotypes defined by several closely linked SNPs. Haplotype association studies are being employed more and more to investigate associations for complex diseases [4]. The generalized linear model (GLM) is a flexible framework that allows for the incorporation of environment factors and interactions between covariates, in which a logistic regression model can be used for binary traits. When rare haplotypes are present, however, the standard log-likelihood approach for GLM could lead to large standard errors for the coefficients of such haplotypes. In fact, the expectation maximization (EM) algorithm, usually employed for estimating such parameters, might not converge at all. Moreover, it would lead to a large degrees of freedom in the haplotype test, and therefore reduced power when there is a large number of haplotypes in an association analysis. On the other hand, GLM with regularization (rGLM) can effectively combat these problems, and in particular, it is applicable to the common disease/rare variant scenario [5].

## Methods

### Data checking

As a quality control measure, we tested for Hardy-Weinberg equilibrium (HWE) in the controls using an exact test. There are 156 and 55 SNPs with HWE *p*-values less than 1.4 × 10^-6 ^and 3 × 10^-6 ^on chromosome 6 and 18, respectively, which are significant (*p *> 0.05) after Bonferroni correction. Moreover, there are 106 and 59 SNPs with monomorphism. We also checked for SNPs with a large amount of missing data, but none of the SNPs were removed based on the criterion of at least 50% missing rate, which was chosen to keep SNPs with a reasonable amount of data in the preliminary step. Thus, a total of 262 and 114 on chromosomes 6 and 18, respectively, were removed either due to the lack of polymorphisms or significant deviations from HWE in the controls. All 2062 samples were used.

### rGLM

To deal with the problems of large standard errors, non-convergence, and reduced power associated with standard GLM likelihood approach, we adopted a statistical learning method that effectively shrinks the coefficients of unassociated haplotypes and reduces the variance of the estimated regression coefficients. One frequently used method for doing this is the use of the LASSO penalty, which shrinks the coefficients of unassociated variables to zeros [6]. This is implemented in the rGLM software [5], which assumes HWE and was used in this study.

rGLM applies the LASSO penalty to a logistic regression model on unphased genotype data. In a case-control study design, the complete data log-likelihood function for individual *i *can be expressed as follows:

where *y*_*i *_and *X*_*i *_(missing) denote the trait value and haplotype of individual *i*, respectively, and β and γ are the logistic regression coefficients and haplotype frequency parameters. Using the LASSO penalty, the complete penalized likelihood function is

where λ is the tuning parameter and *m *is the number of haplotypes. This likelihood function can be maximized by the EM algorithm. To determine the tuning parameter λ, it makes use of a recent result in Zou et al. [7], which shows that the number of non-zero coefficients in a LASSO regression is an unbiased estimate of the degrees of freedom.

### Other analyses

As a preliminary genome scan measure, single-SNP tests were carried out using a genotype-based Fisher's exact test. In addition to the rGLM approach, hapassoc [8] was also employed to test for association on haplotypes as a standard GLM likelihood approach, in which an EM algorithm was used to infer the haplotypes and haplotype effects simultaneously.

## Results

**Figure 1:**
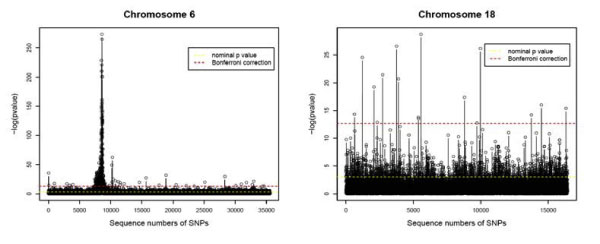
**Single SNP association**. The -log *p-*values for a whole genome analysis by genotype-based exact tests on chromosome 6 and 18. The statistically significant associations at nominal level (yellow line) and at Bonferroni corrected level (red line) are also indicated.

For each single SNP on chromosome 6 and 18, an exact test was carried out based on genotype counts; the *p*-values are shown in Figure 1. There are 424 and 16 statistically significant SNP associations at the Bonferroni corrected level of 5% for chromosome 6 and 18, respectively. It is interesting to note that on chromosome 6, except for 29 SNPs, all the remaining 395 are distributed in a small region, from position 29463092 (7448^th ^SNP: rs238869) to position 33955055 (9113th SNP: rs10947463) (Figure 1, left panel), which covered the HLA-DRB1 allele and most of the HLA region on 6p21. On chromosome 18, 16 significant SNPs were found (Figure 1, right panel), which include SNPs that overlaps with those found in previous studies [2,3].

Because single-SNP tests may be less powerful than haplotype-based tests in many situations, we carried out a two-step haplotype analysis. To reduce the computational demand for genome-wide analysis, in the first step we performed a logistic regression with the LASSO penalty using glmpath [9] using all SNPs assuming an additive model between SNPs and a co-dominant model for each SNP. The missing values were replaced by the most frequent genotypes for the corresponding SNPs. As a result, there were 986 and 249 'tag' SNPs selected on chromosome 6 and 18, respectively. We note that these so called 'tag' SNPs are not the conventional kind that can be considered as the 'proxy' for those not selected. Instead, they are tagged due to their likely association with the disease.

**Figure 2:**
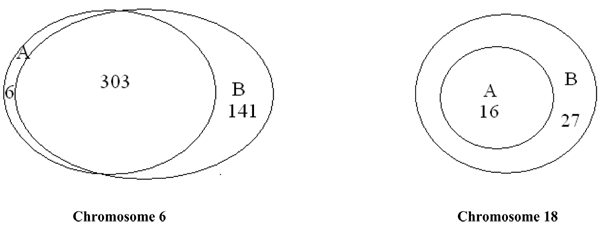
**rGLM vs. hapassoc**. The Venn diagrams for the results from rGLM and hapassoc. A, significant hapassoc tests; B, significant rGLM tests. Left panel and right panel are for chromosomes 6 and 18, respectively.

**Figure 3:**
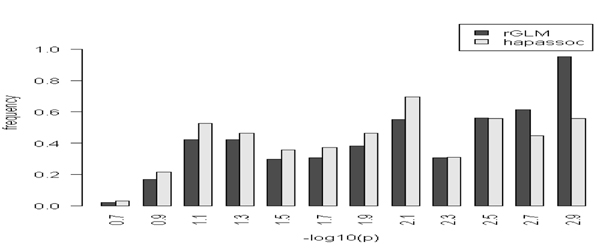
**Frequency distributions of haplotypes**. Histograms of minimum haplotype frequencies (after negative logarithm transformation) of the significant 4-SNP tests identified by rGLM and hapassoc. X-axis denotes the center of each bar and the bar width is 0.2.

In the second step, based on the selected 'tag' SNPs, a four-SNP sliding window was taken to implement the haplotype approaches, rGLM and hapassoc. Due to the existence of rare haplotypes, 64% (out of the total of 1229 four-SNP tests on both chromosomes) of the tests using hapassoc did not converge, whereas rGLM did not encounter such a problem. As shown in Figure 2, for those tests that hapassoc was able to run, analyses yielded 309 and 16 significant results for chromosome 6 and 18, respectively. On the other hand, using the number of nonzero coefficients as an estimate of the degrees of freedom [7], the rGLM gave, for chromosomes 6 and 18, respectively, 444 and 43 significance test results. Indeed, rGLM was able to identify additional significant tests through alleviating the problem of non-convergence. For example, on chromosome 18, all of the 16 significant results identified by hapassoc were included in those found by rGLM. Furthermore, rGLM uncovered 27 additional ones from among the 64% of tests that hapassoc failed to converge. We plotted the minimum frequencies of the significant four-SNP windows (Figure 3), which shows that the distribution of haplotype frequencies among the significant results identified by rGLM indeed contains rarer haplotypes than the distribution representing the frequencies of those identified by hapassoc, reaffirming the value of rGLM for detecting rare variants.

## Discussion

We focused on scanning the SNPs on chromosomes 6 and 18 in our analysis based on evidence from prior studies. For chromosome 6, both single-SNP and haplotype-based approaches identified numerous associated SNPs/haplotypes around the HLA region, solidifying the importance of the HLA region for autoimmune diseases and confirming results from previous studies. Despite many common findings, each of the haplotype-based approaches identified more than 100 four-SNP windows that do not contain any of the significant SNPs selected by single-SNP analysis. This may be explained by the increased power of haplotype analysis, but further investigation is needed.

It is challenging to run whole-genome haplotype-based analysis with only phased-unknown SNP data. To reduce dimensionality, one of the most frequently employed approaches is to find tagging SNPs before embarking on a haplotype-based analysis. We attempted to use the haploview software for such as task. However, the amount of SNP reduction was not sufficient for the subsequent haplotype analysis to be practically feasible -- less than 20% of the SNPs were excluded on each chromosome using haploview. On the other hand, the penalized regression approach as described earlier was able to accomplish this task, leading to the identification of about 3% of SNPs as 'tags'. This remarkable reduction makes our haplotype-based analysis, as well as other computationally intensive approaches for genome-wide studies, possible. However, the loss of information needs to be investigated further.

Using a penalized approach, rGLM shows a good power for detecting the effects of rare haplotypes [5]. Compared to the usual unpenalized GLM, rGLM is powerful and does not encounter the problem of non-convergence. However, the permutation procedure as proposed in Guo and Lin [5] can be too computationally intensive for obtaining *p*-values for studies on a genome-wide scale. Instead, we only obtained *p*-values by permutation and also by chi-square approximations (two different ways, one conservative and one liberal) on a selected subset to gauge whether chi-square approximation will give reasonably good results in this application. We found that for SNP combinations that give small *p*-values (say uncorrected *p *< 0.01), all three methods lead to the same conclusion. Because our interest is in identifying significant haplotypes, we feel that our approximation method for computing the *p*-value is reasonable. However, further research on the appropriateness of such an approximation procedure and whether this will lead to the same type I error rate for hapassoc and rGLM is warranted.

## List of abbreviations used

EM: Expectation maximization; GAW: Genetic Analysis Workshop; GLM: Generalized linear model; HWE: Hardy-Weinberg equilibrium; NARAC: North American Rheumatoid Arthritis Consortium; RA: Rheumatoid arthritis; rGLM: Generalized linear model with regularization; SNP: Single-nucleotide polymorphism.

## Competing interests

The authors declare that they have no competing interests.

## Authors' contributions

WG and SL developed the methodology and devised the analysis scheme. WG carried out the analysis with the assistance of CL. GW and SL drafted the manuscript. All authors read and approved the final manuscript.

## References

[B1] Amos CI Chen WV Seldin MF Remmers E Taylor KE Criswell LA Lee AT Plenge RM Kastner DL Gregersen PK Data for Genetic Analysis Workshop 16 Problem 1, association analysis of rheumatoid arthritis data BMC Proceedings 2009 3 Suppl 7 S2 10.1186/1753-6561-3-s7-s2 PMC2795916 20018009

[B2] Jawaheer D Seldin MF Amos CI Chen WV Shigeta R Etzel C Damle A Xiao X Chen D Lum RF Monteiro J Kern M Criswell LA Albani S Nelson JL Clegg DO Pope R Schroeder HW Jr Bridges SL Jr Pisetsky DS Ward R Kastner DL Wilder RL Pincus T Callahan LF Flemming D Wener MH Gregersen PK North American Rheumatoid Arthritis Consortium Screening the genome for rheumatoid arthritis susceptibility genes: a replication study and combined analysis of 512 multicase families Arthritis Rheum 2003 48 906 916 10.1002/art.10989 12687532

[B3] Osorio Y Fortéa J Bukulmez H Petit-Teixeira E Michou L Pierlot C Cailleau-Moindrault S Lemaire I Lasbleiz S Alibert O Quillet P Bardin T Prum B Olson JM Cornélis F Dense genome-wide linkage analysis of rheumatoid arthritis, including covariates Arthritis Rheum 2004 50 2757 2765 10.1002/art.20458 15457443

[B4] Schaid DJ Evaluating associations of haplotypes with traits Genet Epidemiol 2004 27 348 364 10.1002/gepi.20037 15543638

[B5] Guo W Lin SL Generalized linear modeling with regularization for detecting common disease rare haplotype association Genet Epidemiol 2009 33 308 316 10.1002/gepi.20382 19025789 PMC2752471

[B6] Tibshirani R Regression shrinkage and selection via the lasso J R Stat Soc Series B Stat Methodol 1996 58 267 288

[B7] Zou H Hastie T Tibshirani R On the "degrees of freedom" of the lasso Ann Stat 2007 35 2173 2192 10.1214/009053607000000127

[B8] Burkett K Graham J McNeney B Hapassoc: Software for likelihood inference of trait associations with SNP haplotypes and other attributes J Stat Softw 2006 16 1 19

[B9] Park MY Hastie T L1 regularization path algorithm for generalized linear models J R Stat Soc Series B Stat Methodol 2007 69 659 677 10.1111/j.1467-9868.2007.00607.x

